# Influence of budesonide and fluticasone propionate in the anti-osteoporotic potential in human bone marrow-derived mesenchymal stem cells via stimulation of osteogenic differentiation

**DOI:** 10.1016/j.heliyon.2024.e39475

**Published:** 2024-10-18

**Authors:** Terrence Suministrado Sumague, Abdurahman A. Niazy, Rhodanne Nicole A. Lambarte, Ibrahim A. Nafisah, Arief Gusnanto

**Affiliations:** a Molecular and Cell Biology Laboratory, Prince Naif bin AbdulAziz Health Research Center, King Saud University Medical City, Riyadh, Kingdom of Saudi Arabia; b Department of Oral Medicine and Diagnostic Sciences, College of Dentistry, King Saud University, Riyadh, Kingdom of Saudi Arabia; c Department of Statistics and Operations Research, College of Science, King Saud University, Riyadh, Saudi Arabia; d School of Mathematics, University of Leeds, UK

**Keywords:** Bioinformatics approach, Glucocorticoid compound, Anti-Osteoporosis, Osteogenic differentiation, Mesenchymal stem cells

## Abstract

Osteoporosis is a prevalent bone condition with adverse effects observed in patients undergoing long-term glucocorticoid therapy, resulting in bone demineralization and tissue loss. There has been limited studies on the global response to dexamethasone in terms of comparing its expression profile to other common glucocorticoids during osteogenic differentiation. This study focused on the downregulated gene expression profile of glucocorticoid compounds; dexamethasone, budesonide, and fluticasone propionate, during osteogenic differentiation to elucidate the related target genes and pathways associated with the anti-osteoporotic potential of telomerase-immortalized human bone marrow-derived mesenchymal stem cells using a bioinformatics approach. Based on gene expression microarrays experiments and bioinformatics analysis, several key genes involved in the regulation of osteogenic differentiation and osteoporosis development in mesenchymal stem cells that were targeted by these specific glucocorticoids were determined. Network analysis using GeneCards, OMIM, and CTD databases were performed and osteoporosis-related genes were identified. LIMMA and moderated Welch test R packages were performed to determine significant downregulated differentially expressed genes for each glucocorticoid treatment. A total of 479 (dexamethasone), 84 (budesonide), and 889 (fluticasone propionate) differentially expressed genes were identified for each glucocorticoid, of which 35 common genes overlapped. Enrichment pathway analysis was conducted using Metascape, and protein-protein interaction networks were constructed using the STRING database and Cytoscape software to determine potential target genes involved with osteoporosis. Enrichment pathway analysis revealed genes involved in 3 Reactome pathways namely cytokine signaling in immune system, immune system and the interferon alpha/beta signaling pathways and identified 10 hub genes based on the PPI network to determine potential target pathways associated with osteoporosis. These findings provide preliminary insights into the relationship between the key target genes of dexamethasone, budesonide, and fluticasone propionate, and the pathways associated with regulated osteoporosis metabolism during osteogenic differentiation.

## Graphical abstract

**Figure:**
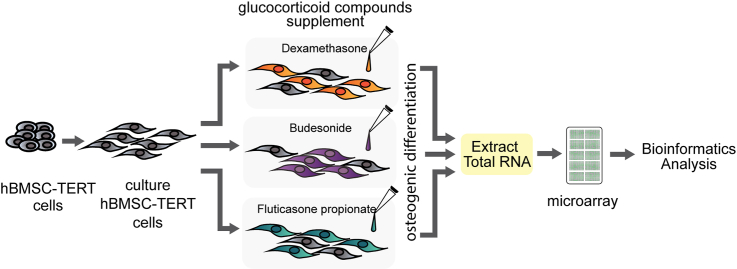

## 1 Introduction

Osteoporosis (OP) induced by chronic glucocorticoid therapy such as dexamethasone (DEX) is a common disorder observed in patients treated with glucocorticoids [1]. However, some reports indicate a strong tendency to strengthen bones rather than cause OP [2]. Generally, short-term glucocorticoid treatment may increase bone strength while long-term therapy reduces bone strength resulting in OP [3]. OP is a condition leading to the progressive loss of bone density as a result of micropores that occur in the bone due to bone tissue loss [4]. The mechanism for glucocorticoid-induced OP is not yet fully understood. However, several studies have shown the positive effects of DEX on osteogenesis [[5], [6], [7]]. DEX is commonly supplemented when culturing mesenchymal stem cells (hMSCs) to induce osteogenic behavior [8]. Multiple osteogenesis-related genes are activated when hMSCs are in contact with DEX [9]. Most studies focused on the upregulation of glucocorticoids in relation to osteogenic potential; however, few studies have addressed its downregulation as a means to study potential causes in OP [[10], [11], [12]]. With glucocorticoids as the leading cause of secondary OP [13], limited research has been conducted to further understand the causes.

Budesonide (BDS) is a widely used glucocorticoid and many studies have shown no decrease in bone density after use [[14], [15], [16]]. Moreover, previous studies such as Sorva et al. [17] observed that during a 12-month period, some bone formation markers have reduced expression and normalized post-treatment. However, other clinical studies have observed insignificant effects and bone density improvement [[18], [19], [20], [21]]. Recent studies have examined the long-term effects of BDS on osteoporotic fractures, which is similarly observed in DEX [22]. Fluticasone propionate (FLT), a common glucocorticoid, is not known to directly affect osteoporosis [23,24]. These two glucocorticoids could be used to further understand the mechanisms by which DEX causes OP. Although DEX, is inexpensive and well-established for its anti-inflammatory properties; it is important to minimize the side effects caused by long-term treatment.

Several studies have analyzed responses to DEX focusing on the epigenetics of osteogenesis. Li et al. [25] have demonstrated that microRNA miR-216a promotes osteogenesis by regulating the c-Cbl-mediated PI3K/AKT pathway with most of the focus on the epigenetic response. Piek et al. [26] studied the global response of DEX with and without vitamin D and have concluded that c-MYC is an enhancer of BMP-2. Furthermore, there has been no extensive gene regulation approach conducted on the global response of DEX to osteogenesis in terms of comparing its global response to other glucocorticoids such as BDS or FLT.

In this study, telomerase-immortalized human mesenchymal stem cells (hMSC-TERT20) were used and supplemented with glucocorticoids. It focuses on the downregulated genes of the whole genome using microarray to identify potential pathways associated with OP using bioinformatics approaches. The results of all three glucocorticoids were compared to help understand the process by which DEX induces OP.

## 2 Materials and methods

### 2.1 Compounds

Budesonide (S2186) and Fluticasone propionate (S1992) were obtained from Selleckchem, Inc. (Houston, TX, USA). Dexamethasone was purchased from Sigma-Aldrich (St. Louis, MO, USA). Compounds were dissolved in ethanol and used at a concentration of 1.0 μM. Control cells were treated with basal medium containing ethanol as vehicle.

### 2.2 In vitro experiments

#### 2.2.1 Cell culture

As a model for human bone marrow stromal stem cells (hBMSCs), a well-characterized hMSC-TERT20 (RRID: CVCL Z018) cell line that has been immortalized by the human telomerase reverse transcriptase gene (hTERT) was used in this study [27,28]. The hMSC-TERT20 cell line was kindly provided by Prof. Moustapha Kassem from the Department of Endocrinology, Odense University Hospital, University of Southern Denmark. Cells were cultured in basal medium of Dulbecco's modified Eagle's medium (DMEM) supplemented with 4500 mg/L D-glucose, 4 mM L-glutamine, 110 mg/L sodium pyruvate, 10 % fetal bovine serum, 1 % penicillin–streptomycin, and 1 % nonessential amino acids and maintained at 37 °C in a humidified 5 % CO_2_ atmosphere. All culture reagents were purchased from Gibco Invitrogen (Carlsbad, CA, USA).

#### 2.2.2 Osteogenic differentiation

The hMSC-TERT20 cells were cultured to 80 % confluency and exposed to osteogenic induction medium (DMEM containing 10 % FBS, 1 % penicillin-streptomycin, 50.0 μg/mL L-ascorbic acid (Vitamin C; Winlab Scientific Ltd, West Sussex, United Kingdom), 10.0 mM β-glycerophosphate (β-GP; Sigma-Aldrich, St. Louis, MO, USA) and 10.0 nM cholecalciferol ((+)-Vitamin D3; Sigma Aldrich, St. Louis, MO, USA), supplemented with the glucocorticoid compounds DEX, BDS or FLT at 1.0 μM. The osteogenic medium and its supplementation were changed every 3 days and the experiments were terminated after 10 days of culture.

#### 2.2.3 Total RNA extraction

Total RNA was isolated from cell pellets collected after 10 days of osteogenic differentiation using the Total RNA Purification Kit (Norgen Biotek Corp., Thorold, ON, Canada, https://norgenbiotek.com/) according to the manufacturer's protocol. The quality and concentrations of total RNA were measured using the Eppendorf BioSpectrometer® basic (Hamburg, Germany).

#### 2.2.4 Microarray preparation, hybridization, and scanning

Preparation, hybridization and processing of microarray RNA samples were performed at the Microarray Core Facility of Stem Cell Unit, Department of Anatomy, College of Medicine located at the King Saud University, Riyadh, Saudi Arabia. A low input QuickAmp Labeling Kit (Agilent Technologies, Santa Clara, CA, http://www.agilent.com) was used to label 150 ng of total RNA then hybridized to the SurePrint G3 Human GE 8 × 60K (Agilent Technologies) microarray chip. Probe signal intensities were scanned using Agilent Technologies Scanner G2505C US10493856 and processed with Feature Extraction Software 11.0 (Agilent Technologies, Palo Alto, CA, U.S.A.).

### 2.3 Bioinformatics analysis

#### 2.3.1 Acquisition and screening of target OP-related genes

The potential target genes related to OP were retrieved from GeneCards (https://www.GeneCards.org/), Online Mendelian Inheritance in Man (https://www.omim.org/), and Comparative Toxicogenomics Database (https://ctdbase.org/) databases using the “osteoporosis” keyword. The obtained lists were filtered to remove duplicate genes and then integrated as the target OP-related genes.

#### 2.3.2 Microarray data pre-processing, normalization and analysis

To prepare the data for statistical analysis, the raw microarray data were pre-processed and normalized using the R package Linear Models for Microarray Data (LIMMA) version 3.42 [29] (available online: http://www.bioconductor.org/). Background correction of signal intensities using “normexp” method, which models the background noise using a normal distribution and signal using an exponential distribution. The quantile normalization method was used to ensure the normalization of values across all arrays data. Differential expression was analyzed using the moderated Welch *t*-test to deal with potential unbalanced group variance and safeguard against false positives [30], which is implemented in the R package Moderated Welch Test (MWT) version 0.2.7. Differentially expressed genes were selected if the fold change (FC) is greater than two and the MWT P-value is less than 0.05 after adjusted for false discovery rate correction [31]. Specifically, the study analyzed the differentially expressed genes (DEGs) that were significantly downregulated with respect to vehicle control-treated-BMSCs of DEX, BDS and FLT that potentially influence the osteogenic differentiation associated to osteoporosis.

#### 2.3.3 Screening of target GC-OP intersected genes and PPI network construction and visualization

Each list of genes from the GCs DEGs and target OP-related genes were input in InteractiVenn (https://www.interactivenn.net/) to create Venn diagrams and to obtain crossover potential target genes. The common DEGs among DEX-, BDS-, and FLT-OP were input in the STRING (Search Tool for the Retrieval of Interacting Genes database; version 11.0; https://string-db.org/) database [32] using “*Homo sapiens*”, and 0.4 for minimum interaction threshold, to determine functional enrichment [30,33,34]. The generated PPI network was further analyzed with Cytoscape [35] using the cytoHubba version 0.1 using the default ranking method to determine the top 10 nodes ranked among the hub genes; and the NetworkAnalyzer version 4.5.0 to further calculate the network topology [35]. The style of the final set of genes were edited using according the MCC rank and calculated degree.

#### 2.3.4 Gene ontology and pathway enrichment analysis of target osteoporosis-related genes

Gene ontology (GO) and enrichment pathway analysis were performed using Metascape (Release 3.5; 2019-03-01; http://metascape.org), a web-based tool for gene annotation and analysis resource [36]. Metascape integrates different robust resources, including Kyoto Encyclopedia of Genes and Genomes (KEGG; Release 94.0, 2020-04-01; https://www.genome.jp/kegg), Gene Ontology Biological Processes (GO:BP) (GO; Release 2020-05-02; http://geneontology.org/), Reactome Gene Sets (version 71; https://reactome.org) and Molecular Signatures Database (MSigDB; version 7.1; https://www.gsea-msigdb.org/gsea/msigdb/index.jsp). Gene set enrichment analysis was performed using the overrepresentation test and the p-value is calculated based on the cumulative hypergeometric distribution. Significant enriched GO terms were considered if the P-value is less than 0.01 after FDR adjustment [31], a minimum of three matched genes per gene set, an enrichment factor >1.5 (the enrichment factor is the ratio between observed count and the count expected by chance) and clustered based on membership similarity. An enrichment factor 2 indicated the most statistically significant term within a cluster and was chosen to represent the cluster. Kappa scores were used as the similarity metric when performing hierarchical clustering on the enriched terms, and sub-trees with a similarity of >0.3 were considered a cluster [36].

#### 2.3.5 Molecular docking verification of key target and GCs components

The GCs compound chemical structure were downloaded from the PubChem Compound database (https://pubchem.ncbi.nlm.nih.gov/) (PubChem ID: 5743) for DEX, (PubChem ID: 5281004) for BDS and (PubChem ID: 444036). The ligand geometry was optimized using Avogadro 1.2.0 (https://avogadro.cc) and output to mol2 format. The predicted target OP-related genes were further evaluated by molecular docking. The crystal structures of matrix metallopeptidase 1 (MPP1) (PDB ID: 3SHI), chitinase 3 like 1 (CHI3L1) (PDB ID: 8R41), and interleukin 11 (IL11) (PDB: 4MHL) were extracted from RSCSB Protein Data Bank (www.rcsb.org/) [37]. AutoDockTools 1.5.7 software was used to read target protein files, hydrogenated, calculated charges and exported. The blind docking was perform using AutoDock Vina 1.1.2 [38]. The grid box was center on the geometric center; (x = 18.441, y = −15.662, z = 1.399) for MPP1; (x = −40.739, y = −10.415, z = 20.640) for 3SHI; and (x = 17.927, y = 5.086, z = 46.587) for IL11. The grid box was size was set to 126 × 126 × 126 Å to sufficiently cover the entire protein surface for unbiased potential binding site exploration. The parameter value of grid spacing was set to 1 Å, exhaustiveness was set to 32, and the binding modes was set to 20 to provide thorough sampling of large search space for each ligand. The diverse binding poses were captured using 10 kcal/mol energy range parameter. The lower the binding affinity scores indicate the most stable binding between the ligand and receptor. Finally, the docking result complex was computed using the PyMol 3.0.3 software, visualized and further analyzed using Discovery Studio 2024 Client.

## 3 Results

### 3.1 Microarray data information and identification of DEGs among glucocorticoid-treated hBMSCs

**Fig. 1:**
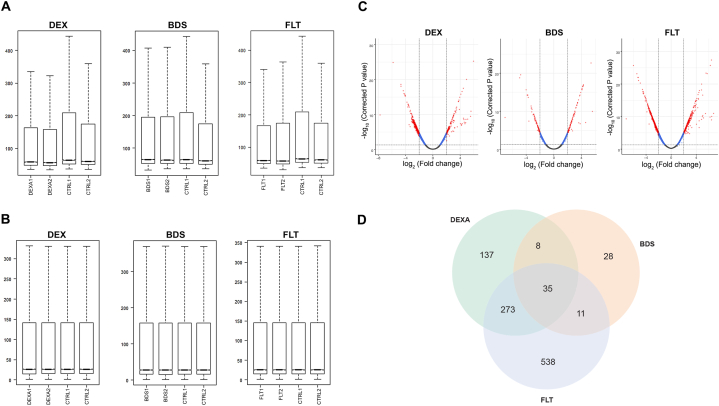
Microarray data pre-processing, normalization and analysis. The distribution of gene expressions from glucocorticoid-treated hBMSCs datasets A. before and B. after normalization. Dataset signal distributions plots calculated in R MWT software package, y-axis represents FDR value and x-axis is coefficient fold-change, where coefficient < −2.0 and FDR <0.05 is significant. C. Volcano plot showing the up and downregulated DEGs among GCs. Red color represents significant, blue color and gray color indicates non-significant DEG. D. Venn diagram analysis depicting the overlap of downregulated differentially expressed genes among DEX, BDS and FLT. Overall, 35 common genes from 1452 total genes. (For interpretation of the references to color in this figure legend, the reader is referred to the Web version of this article.)

The glucocorticoid-treated hBMSCs expression microarray datasets of DEX, BDS and FLT were standardized and screened by LIMMA package, the results of the normalization can be seen in Fig. 1A. Normalization was utilized to correct the distribution between arrays and enabled a direct comparison between them as shown in Fig. 1B. Using MWT package in R, we identified a total of 1452 unique differentially expressed genes. Respectively, 479, 84, and 899 genes that are downregulated in samples that are treated with DEX, BDS and FLT, relative to the untreated ones (Fig. 1C and Supplementary File 1). A total of 35 genes are similar among the treated samples. Indeed, there were more differentially expressed genes that were unique to DEX than in the BDS, 137 vs 28, respectively. Interestingly, there were 538 differentially expressed genes unique in FLT compared with DEX indicating potential genes or associated pathways in OP (Fig. 1D.). The lists of top 20 downregulated DEGs with the lowest FDR value of each treated samples are shown in Table 1.

**Table 1:** List of top 20 downregulated differentially expressed genes for each glucocorticoid-treated hBMSCs.

Gene	Gene full name	MWT	FC	P-value	FDR
Dexamethasone					
*MMP1*	matrix metallopeptidase 1	−16.24	−5.83	6.34 × 10^−30^	1.61 × 10^−25^
*CH25H*	cholesterol 25-hydroxylase	−13.03	−4.68	2.29 × 10^−23^	2.90 × 10^−19^
*SFRP2*	secreted frizzled-related protein 2	−12.74	−4.67	9.88 × 10^−23^	1.00 × 10^−18^
*FNDC1*	fibronectin type III domain containing 1	−12.08	−4.33	2.60 × 10^−21^	1.88 × 10^−17^
*RARRES2*	retinoic acid receptor responder 2	−11.65	−4.23	2.22 × 10^−20^	1.25 × 10^−16^
*SLCO4A1*	solute carrier organic anion transporter family, member 4A1	−11.55	−4.14	3.45 × 10^−20^	1.75 × 10^−16^
*DDIT4L*	DNA-damage-inducible transcript 4-like	−11.37	−4.07	8.38 × 10^−20^	3.54 × 10^−16^
*NKD2*	naked cuticle homolog 2 (Drosophila)	−11.22	−4.04	1.89 × 10^−19^	6.84 × 10^−16^
*BST2*	bone marrow stromal cell antigen 2	−10.38	−3.79	1.31 × 10^−17^	3.49 × 10^−14^
*CNIH3*	cornichon family AMPA receptor auxiliary protein 3	−10.11	−3.62	4.98 × 10^−17^	1.26 × 10^−13^
*COL14A1*	collagen, type XIV, alpha 1	−10.05	−3.61	7.11 × 10^−17^	1.72 × 10^−13^
*MYBPH*	myosin binding protein H	−9.94	−3.57	1.18 × 10^−16^	2.72 × 10^−13^
*CHI3L1*	chitinase 3-like 1 (cartilage glycoprotein-39)	−9.84	−3.53	1.89 × 10^−16^	4.17 × 10^−13^
*CLU*	clusterin	−9.71	−3.67	3.91 × 10^−16^	8.27 × 10^−13^
*ADRA2A*	adrenoceptor alpha 2A	−9.67	−3.46	4.56 × 10^−16^	8.89 × 10^−13^
*PTGDS*	prostaglandin D2 synthase 21 kDa (brain)	−9.22	−3.30	4.43 × 10^−15^	7.49 × 10^−12^
*MFAP2*	microfibrillar-associated protein 2	−9.04	−3.34	1.14 × 10^−14^	1.65 × 10^−11^
*FBLN1*	fibulin 1	−8.79	−3.26	4.10 × 10^−14^	5.54 × 10^−11^
*IER3*	immediate early response 3	−8.79	−3.19	4.15 × 10^−14^	5.54 × 10^−11^
*ARL4C*	ARF like GTPase 4C	−8.73	−3.12	5.37 × 10^−14^	6.85 × 10^−14^
**Budesonide**					
*MMP1*	matrix metallopeptidase 1	−13.62	−4.79	1.24 × 10^−24^	3.13 × 10^−20^
*DDIT4L*	DNA-damage-inducible transcript 4-like	−11.42	−4.00	6.18 × 10^−20^	2.69 × 10^−16^
*RARRES2*	retinoic acid receptor responder (tazarotene induced) 2	−10.74	−3.86	2.01 × 10^−18^	6.56 × 10^−15^
*DDIT4L*	DNA-damage-inducible transcript 4-like	−10.72	−3.78	2.20 × 10^−18^	6.56 × 10^−15^
*CH25H*	secreted frizzled-related protein 2	−10.74	−3.77	1.87 × 10^−18^	6.56 × 10^−15^
*SNORA19*	small nucleolar RNA, H/ACA box 19	−10.43	−3.69	9.97 × 10^−18^	2.66 × 10^−14^
*MYBPH*	myosin binding protein H	−9.33	−3.28	2.59 × 10^−15^	4.87 × 10^−12^
*SNORA15*	small nucleolar RNA, H/ACA box 15	−9.08	−3.21	9.39 × 10^−15^	1.64 × 10^−11^
*CNIH3*	cornichon family AMPA receptor auxiliary protein 3	−8.91	−3.15	2.28 × 10^−14^	3.61 × 10^−11^
*IFI27*	interferon, alpha-inducible protein 27	−8.28	−2.90	5.30 × 10^−13^	6.72 × 10^−10^
*BST2*	bone marrow stromal cell antigen 2	−8.27	−2.90	5.53 × 10^−13^	6.85 × 10^−10^
*IFI6*	interferon, alpha-inducible protein 6	−8.02	−2.81	1.95 × 10^−12^	2.02 × 10^−09^
*SNORA27*	small nucleolar RNA, H/ACA box 27	−7.98	−2.83	2.33 × 10^−12^	2.23 × 10^−09^
*SLCO4A1*	solute carrier organic anion transporter family, member 4A1	−7.97	−2.79	2.32 × 10^−12^	2.23 × 10^−09^
*CHI3L1*	chitinase 3-like 1 (cartilage glycoprotein-39)	−7.85	−2.76	4.40 × 10^−12^	4.13 × 10^−09^
*IL11*	interleukin 11	−7.75	−2.72	6.97 × 10^−12^	6.10 × 10^−09^
*ARL4C*	ADP-ribosylation factor-like 4C	−7.69	−2.70	9.96 × 10^−12^	8.56 × 10^−09^
*SNORA81*	small nucleolar RNA, H/ACA box 81	−7.62	−2.79	1.42 × 10^−11^	1.18 × 10^−08^
*SLC37A2*	solute carrier family 37	−7.59	−2.76	1.63 × 10^−11^	1.31 × 10^−08^
**Fluticasone propionate**					
*MMP1*	matrix metallopeptidase 1	−16.51	−6.01	1.90 × 10^−30^	3.22 × 10^−26^
*MYBPH*	myosin binding protein H	−15.03	−5.46	1.33 × 10^−27^	1.13 × 10^−23^
*DDIT4L*	DNA-damage-inducible transcript 4-like	−14.45	−5.28	2.41 × 10^−26^	1.53 × 10^−22^
*CHI3L1*	chitinase 3-like 1 (cartilage glycoprotein-39)	−14.47	−5.26	2.27 × 10^−26^	1.53 × 10^−22^
*SFRP2*	secreted frizzled-related protein 2	−13.98	−5.19	2.36 × 10^−25^	1.33 × 10^−21^
*CH25H*	cholesterol 25-hydroxylase	−13.54	−4.91	1.62 × 10^−24^	8.24 × 10^−21^
*DDIT4L*	DNA-damage-inducible transcript 4-like	−12.59	−4.57	1.73 × 10^−22^	7.31 × 10^−19^
*PTGDS*	prostaglandin D2 synthase 21 kDa (brain)	−12.28	−4.50	9.01 × 10^−22^	3.05 × 10^−18^
*COL14A1*	collagen, type XIV, alpha 1	−12.22	−4.45	1.27 × 10^−21^	4.01 × 10^−18^
*FBLN1*	fibulin 1	−12.00	−4.47	3.67 × 10^−21^	9.79 × 10^−18^
*C11orf96*	chromosome 11 open reading frame 96	−12.01	−4.37	3.63 × 10^−21^	9.79 × 10^−18^
*RARRES2*	retinoic acid receptor responder 2	−11.88	−4.42	6.25 × 10^−21^	1.59 × 10^−17^
*ADRA2A*	adrenoceptor alpha 2A	−11.55	−4.19	3.20 × 10^−20^	7.06 × 10^−17^
*SLCO4A1*	solute carrier organic anion transporter family, member 4A1	−11.46	−4.18	5.68 × 10^−20^	1.20 × 10^−16^
*FNDC1*	fibronectin type III domain containing 1	−11.30	−4.11	1.28 × 10^−19^	2.59 × 10^−16^
*CNIH3*	cornichon family AMPA receptor auxiliary protein 3	−11.10	−4.01	3.07 × 10^−19^	5.37 × 10^−16^
*FAM134B*	family with sequence similarity 134, member B	−11.04	−4.00	4.46 × 10^−19^	7.29 × 10^−16^
*ZNF581*	zinc finger protein 581	−10.84	−3.98	1.24 × 10^−18^	1.91 × 10^−15^
*CLU*	clusterin	−10.77	−4.13	1.78 × 10^−18^	2.48 × 10^−15^
*LY6E*	lymphocyte antigen 6 complex, locus E	−10.77	−3.98	1.81 × 10^−18^	2.48 × 10^−15^

### 3.2 Analysis of downregulated OP-related DEGs among glucocorticoid-treated hBMSCs

The analysis consisted of examining the differences among gene expression profiles of treated hBMSCs with DEX, BDS and FLT compounds. Lowest number of DEGs is observed in BDS, while DEX and FLT reported greater number of DEGs. Fig. 1D Venn diagrams depicts 21 similar genes among the glucocorticoid-treated hBMSCs in terms of the total downregulated gene sets. Table 2 shows the summary of gene similarities and its expression levels wherein *ADRA2A, CHI3L1, COL14A1, MMP1, SFRP2, and TNFAIP6* genes present higher significance in FLT compared with DEX and BDS.

**Table 2:** Gene similarity identified from downregulated OP-related DEGs sets in glucocorticoid-treated hBMSCs.

Gene	Gene full name	Fold change		
		DEX	BDS	FLT
*ACKR3*	atypical chemokine receptor 3 [HGNC:23692]	−2.94	−2.15	−2.07
*ADAMTSL2*	ADAMTS like 2 [HGNC:14631]	−2.17	−2.36	−2.08
*ADRA2A*	adrenoceptor alpha 2A [HGNC:281]	−3.46	−2.34	−4.19
*ARL4C*	ADP ribosylation factor like GTPase 4C [HGNC:698]	−3.12	−2.70	−2.84
*BST2*	bone marrow stromal cell antigen 2 [HGNC:1119]	−3.39	−2.90	−2.58
*CH25H*	cholesterol 25-hydroxylase [HGNC:1907]	−4.68	−3.77	−4.91
*CHI3L1*	chitinase 3 like 1 [HGNC:1932]	−3.53	−2.76	−5.26
*CNIH3*	cornichon family AMPA receptor auxiliary protein 3 [HGNC:26802]	−3.62	−3.15	−4.01
*COL14A1*	collagen type XIV alpha 1 chain [HGNC:2191]	−3.12	−2.22	−3.65
*FNDC1*	fibronectin type III domain containing 1 [HGNC:21184]	−4.33	−2.51	−4.11
*FOXQ1*	forkhead box Q1 [HGNC:20951]	−2.94	−2.25	−2.06
*GPER1*	G protein-coupled estrogen receptor 1 [HGNC:4485]	−2.74	−2.15	−2.50
*IFI27*	interferon alpha inducible protein 27 [HGNC:5397]	−2.81	−2.90	−2.01
*IFI6*	interferon alpha inducible protein 6 [HGNC:4054]	−2.96	−2.81	−2.08
*IL11*	interleukin 11 [HGNC:5966]	−3.08	−2.72	−2.42
*MMP1*	matrix metallopeptidase 1 [HGNC:7155]	−5.83	−4.79	−6.01
*PTGDS*	prostaglandin D2 synthase [HGNC:9592]	−3.30	−2.12	−4.50
*RARRES2*	retinoic acid receptor responder 2 [HGNC:9868]	−4.23	−3.86	−4.42
*SAA2*	serum amyloid A2 [HGNC:10514]	−2.35	−2.23	−2.87
*SERTAD4*	SERTA domain containing 4 [HGNC:25236]	−2.14	−2.03	−2.18
*SFRP2*	secreted frizzled related protein 2 [HGNC:10777]	−4.67	−2.63	−5.19
*SLCO4A1*	solute carrier organic anion transporter family member 4A1 [HGNC:10953]	−4.14	−2.79	−4.18
*SULF2*	sulfatase 2 [HGNC:20392]	−3.04	−2.32	−3.25
*TNFAIP6*	TNF alpha induced protein 6 [HGNC:11898]	−2.18	−2.04	−2.96

### 3.3 Pathway and process enrichment analysis of DEGS from treated hBMSCs

**Fig. 2:**
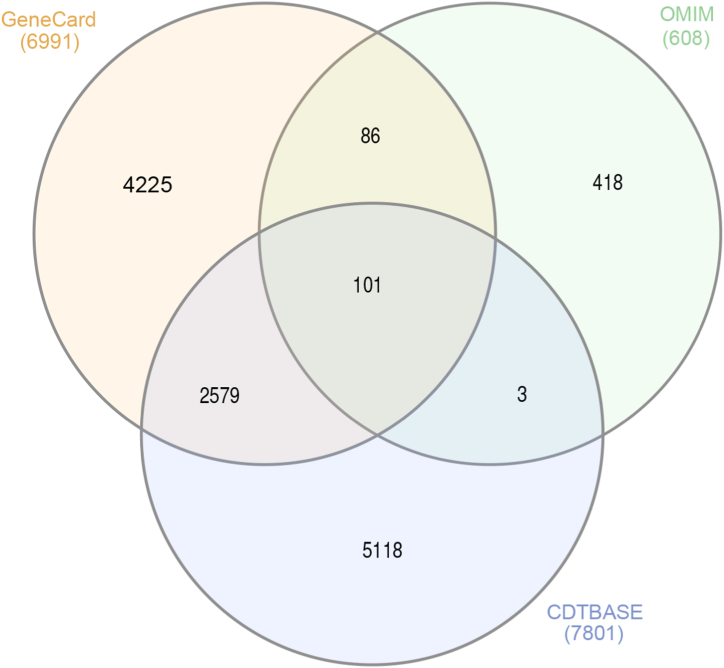
The Venn diagram of OP target genes based on database analysis.

As shown in Fig. 2, the number of OP-related genes were obtained from GeneCards, OMIM and CTD databases was 6991, 608 and 7801 respectively. The gene list of three databases were merged and removed duplicate genes to obtain the list of OP-associated genes.

**Fig. 3:**
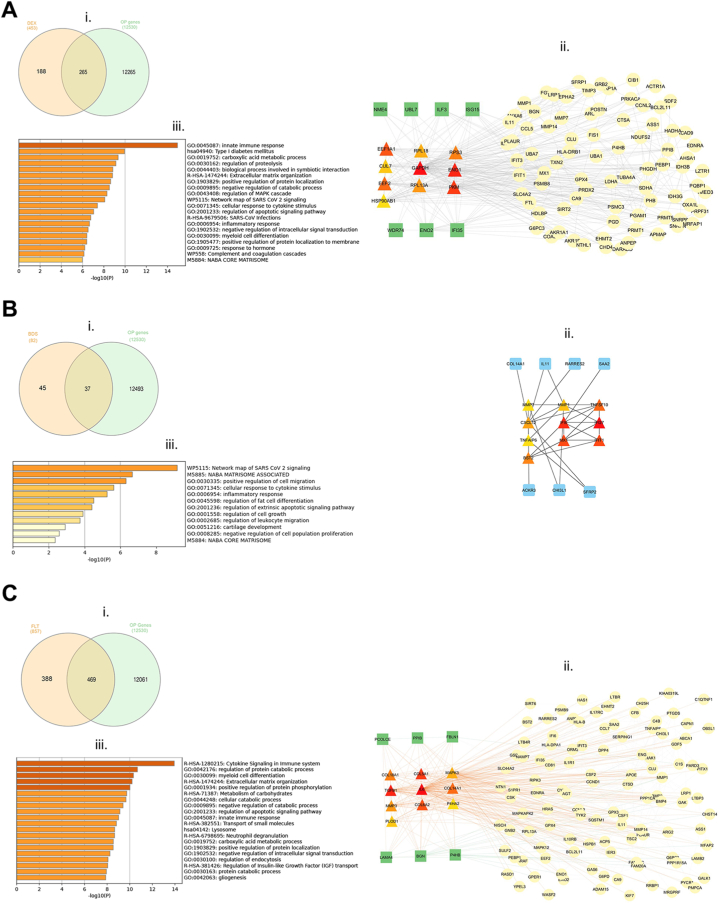
Visualization of pathway and process enrichment analysis of OP-related DEGS (A) DEX- (B) BDS- and (C) FLT-treated hBMSCs. i. Venn diagram of GCs and OP-related intersection genes; ii. A PPI network of GC OP genes. Triangle represent main hub genes; green squares and yellow circles represent first-neighbor node of main hub genes. Main hub genes color intensities indicate node hub MCC ranking score; iii. Top enriched terms across differentially expressed genes, colored by P values represented as heatmap. (For interpretation of the references to color in this figure legend, the reader is referred to the Web version of this article.)

The identified significant downregulated DEGs were analyzed using Metascape enrichment analysis shown in heatmap diagram (Fig. 3, Supplementary File 2) and the top 20 enriched GO terms which were selected are presented in Table 3.

**Table 3:** List of top 20 enriched GO terms from downregulated OP-related DEGs of GCs-treated hBMSCs. Column (count) represent number of genes, percent sign (%) refers to percentage of all of the genes that are found in the given ontology term, log10(P) is the p-value in log base 10 and log10(q) is the calculated multi-test adjusted p-value in log base 10.

GO	Category	Description	Count	%	Log_10_(P)	Log_10_(q)
Dexamethasone						
GO:0045087	GO Biological Processes	innate immune response	35	13.21	−14.94	−10.59
hsa04940	KEGG Pathway	Type I diabetes mellitus	9	3.40	−9.95	−6.30
GO:0019752	GO Biological Processes	carboxylic acid metabolic process	28	10.57	−9.35	−5.90
GO:0030162	GO Biological Processes	regulation of proteolysis	24	9.06	−9.13	−5.74
GO:0044403	GO Biological Processes	biological process involved in symbiotic interaction	16	6.04	−8.85	−5.65
R-HSA-1474244	Reactome Gene Sets	Extracellular matrix organization	17	6.42	−8.82	−5.65
GO:1903829	GO Biological Processes	positive regulation of protein localization	21	7.92	−8.64	−5.50
GO:0009895	GO Biological Processes	negative regulation of catabolic process	18	6.79	−8.52	−5.44
GO:0043408	GO Biological Processes	regulation of MAPK cascade	24	9.06	−8.29	−5.27
WP5115	WikiPathways	Network map of SARS CoV 2 signaling	15	5.66	−8.09	−5.12
GO:0071345	GO Biological Processes	cellular response to cytokine stimulus	24	9.06	−7.40	−4.56
GO:2001233	GO Biological Processes	regulation of apoptotic signaling pathway	17	6.42	−7.04	−4.25
R-HSA-9679506	Reactome Gene Sets	SARS-CoV Infections	17	6.42	−6.77	−4.08
GO:0006954	GO Biological Processes	inflammatory response	20	7.55	−6.65	−4.00
GO:1902532	GO Biological Processes	negative regulation of intracellular signal transduction	22	8.30	−6.52	−3.96
GO:0030099	GO Biological Processes	myeloid cell differentiation	14	5.28	−6.40	−3.89
GO:1905477	GO Biological Processes	positive regulation of protein localization to membrane	9	3.40	−6.39	−3.89
GO:0009725	GO Biological Processes	response to hormone	23	8.68	−6.21	−3.75
WP558	WikiPathways	Complement and coagulation cascades	7	2.64	−6.13	−3.71
M5884	Canonical Pathways	NABA CORE MATRISOME	13	4.91	−5.98	−3.59
**Budesonide**						
WP5115	WikiPathways	Network map of SARS CoV 2 signaling	8	21.62	−9.17	−4.83
M5885	Canonical Pathways	NABA MATRISOME ASSOCIATED	9	24.32	−6.65	−2.79
GO:0030335	GO Biological Processes	positive regulation of cell migration	8	21.62	−6.31	−2.65
GO:0071345	GO Biological Processes	cellular response to cytokine stimulus	8	21.62	−5.62	−2.31
GO:0006954	GO Biological Processes	inflammatory response	7	18.92	−5.25	−2.06
GO:0045598	GO Biological Processes	regulation of fat cell differentiation	4	10.81	−4.51	−1.53
GO:2001236	GO Biological Processes	regulation of extrinsic apoptotic signaling pathway	4	10.81	−4.40	−1.47
GO:0001558	GO Biological Processes	regulation of cell growth	5	13.51	−3.91	−1.19
GO:0002685	GO Biological Processes	regulation of leukocyte migration	4	10.81	−3.74	−1.07
GO:0051216	GO Biological Processes	cartilage development	3	8.11	−2.90	−0.51
GO:0008285	GO Biological Processes	negative regulation of cell population proliferation	5	13.51	−2.59	−0.27
M5884	Canonical Pathways	NABA CORE MATRISOME	3	8.11	−2.34	−0.05
**Fluticasone propionate**						
R-HSA-1280215	Reactome Gene Sets	Cytokine Signaling in Immune system	46	9.85	−13.94	−9.60
GO:0042176	GO Biological Processes	regulation of protein catabolic process	27	5.78	−10.68	−6.64
GO:0030099	GO Biological Processes	myeloid cell differentiation	24	5.14	−10.33	−6.47
R-HSA-1474244	Reactome Gene Sets	Extracellular matrix organization	24	5.14	−10.21	−6.47
GO:0001934	GO Biological Processes	positive regulation of protein phosphorylation	33	7.07	−10.02	−6.37
R-HSA-71387	Reactome Gene Sets	Metabolism of carbohydrates	23	4.93	−9.73	−6.23
GO:0044248	GO Biological Processes	cellular catabolic process	39	8.35	−9.67	−6.23
GO:0009895	GO Biological Processes	negative regulation of catabolic process	25	5.35	−9.41	−6.11
GO:2001233	GO Biological Processes	regulation of apoptotic signaling pathway	26	5.57	−9.13	−5.90
GO:0045087	GO Biological Processes	innate immune response	37	7.92	−9.02	−5.85
R-HSA-382551	Reactome Gene Sets	Transport of small molecules	36	7.71	−8.82	−5.68
hsa04142	KEGG Pathway	Lysosome	15	3.21	−8.67	−5.59
R-HSA-6798695	Reactome Gene Sets	Neutrophil degranulation	28	6.00	−8.66	−5.59
GO:0019752	GO Biological Processes	carboxylic acid metabolic process	37	7.92	−8.55	−5.52
GO:1903829	GO Biological Processes	positive regulation of protein localization	28	6.00	−8.54	−5.52
GO:1902532	GO Biological Processes	negative regulation of intracellular signal transduction	34	7.28	−8.25	−5.27
GO:0030100	GO Biological Processes	regulation of endocytosis	21	4.50	−8.08	−5.13
R-HSA-381426	Reactome Gene Sets	Regulation of Insulin-like Growth Factor (IGF) transport and uptake by Insulin-like Growth Factor Binding Proteins (IGFBPs)	14	3.00	−8.05	−5.12
GO:0030163	GO Biological Processes	protein catabolic process	34	7.28	−7.94	−5.03
GO:0042063	GO Biological Processes	gliogenesis	20	4.28	−7.88	−4.99

The downregulated DEGs were analyzed using Venn diagram to determine the overlapping genes of GCs and OP-related (Fig. 3A–C, i.), 265, 37, and 469 for DEX, BDS, and FLT respectively. In order to clarify the potential anti-osteoporotic mechanism of glucocorticoid compounds in hBMSCs, the PPI network analysis (Fig. 3A–C ii.) were mapped in order to determine the functional classifications and generate functional analysis of interacted genes and subnetworks. The obtained genes were input into the Cytoscape STRING database to construct the PPI network. As a result, DEX consist of 98 nodes and 171 interaction edges with PPI enrichment p-value of < 1.0 × 10^-^^1^^6^ and average local clustering coefficient = 0.395 (Fig. 3A, ii.); 17 nodes and 32 interaction edges with PPI enrichment p-value of < 1.0 × 10^–1^^6^ and avg. local clustering coefficient = 0.424 in BDS (Fig. 3B, ii.); and 127 nodes and 585 interaction edges with PPI enrichment p-value of < 1.0 × 10^-16^ and average local clustering coefficient = 0.347 in FLT (Fig. 3C, ii.). Metascape enrichment analysis shown in heatmap diagram (Fig. 3A–C iii., Supplementary File 2) and the top 20 enriched GO terms which were selected are presented in Table 3.

### 3.4 Protein–protein interaction network analysis for anti-osteoporotic potential from glucocorticoid treated-hBMSCs

**Fig. 4:**
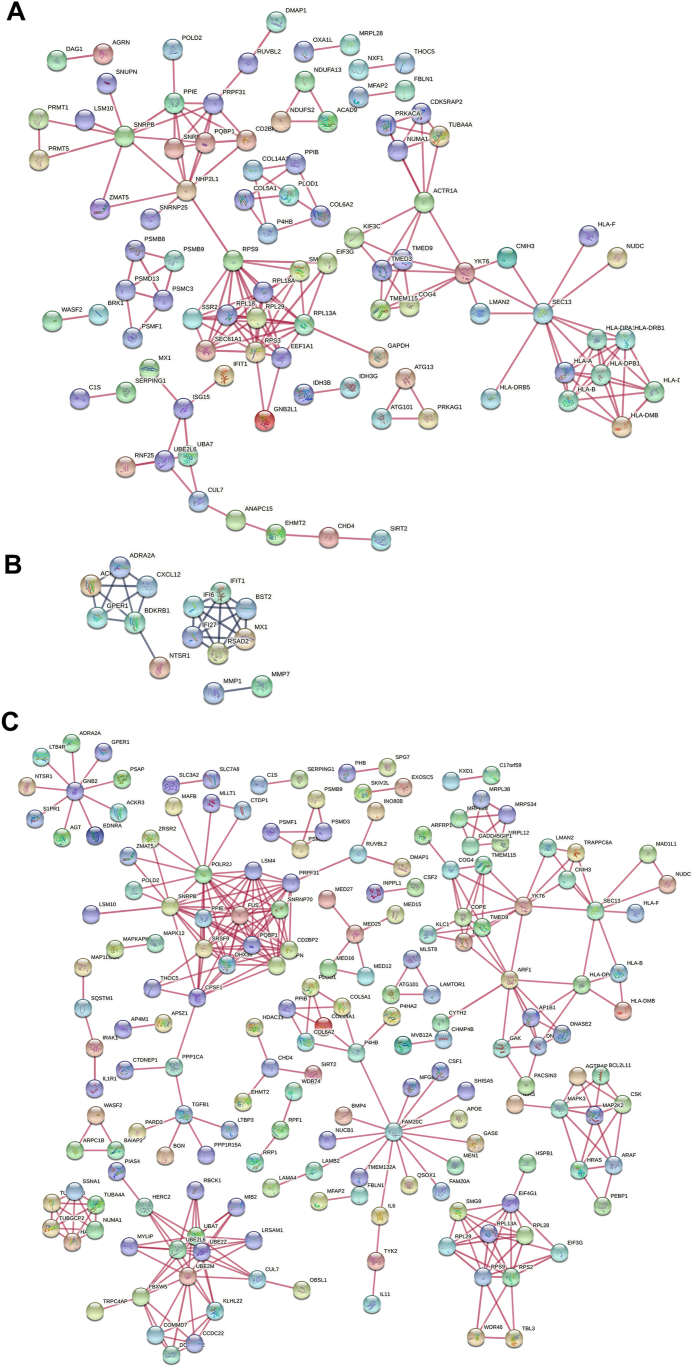
Protein–protein interaction network. Circles represent genes, lines represent the interaction of proteins between genes, and the results within the circle represent the structure of proteins. The downregulated proteins among treated-hBMSCs were analyzed by STRING and network was built using ‘highest confidence’ (0.900) interaction score. Each node represents a protein, line thickness indicates the strength of data support, and the edges indicate that the proteins are part of a physical complex.

In order to clarify the potential anti-osteoporotic mechanism of glucocorticoid compounds in hBMSCs, significant downregulated differentially expressed genes from DEX, BDS and FLT treatment were mapped in order to determine the functional classifications and generate functional analysis of interacted subnetworks. The obtained DEGs were introduced into the STRING online database to construct the PPI network. As a result, DEX consist of 402 nodes and 185 interaction edges with PPI enrichment p-value of 1.86 × 10^−6^ and average local clustering coefficient = 0.181 (Fig. 4A); 50 nodes and 27 interaction edges with PPI enrichment p-value of 7.66 × 10^−15^ and avg. local clustering coefficient = 0.272 in BDS (Fig. 4B); and 740 nodes and 342 interaction edges with PPI enrichment p-value of 0.192 and average local clustering coefficient = 0.191 in FLT (Fig. 4C).

### 3.5 OP-related genes, network construction, enrichment analysis, and visualization

**Fig. 5:**
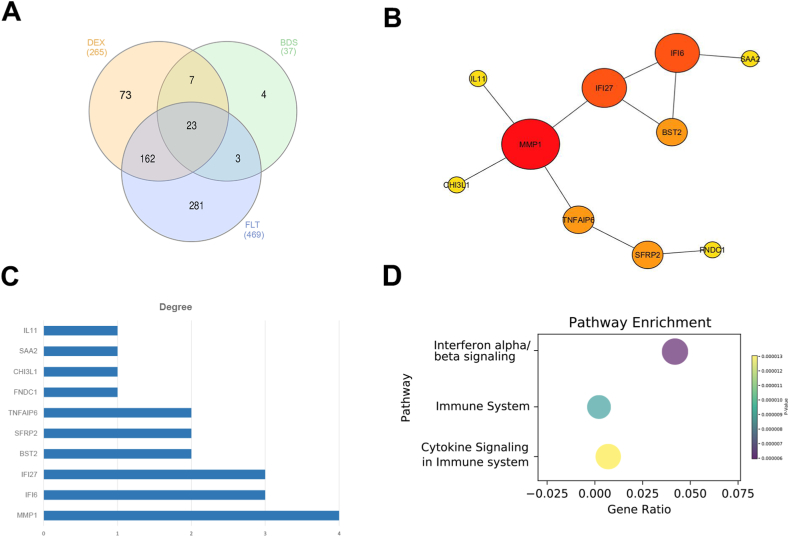
Protein–protein interaction network construction of OP-related genes. (A) Venn diagram of GC common genes matched from OP-related genes. (B) Common GCs gene hubs. Circle size represents the node degree and color intensity represents the hub ranking score. (C) PPI hub genes calculated node degree (D) Bubble plot of function enrichment using STRING. (For interpretation of the references to color in this figure legend, the reader is referred to the Web version of this article.)

A total of 21 common target genes were obtained from the Venn diagram (Fig. 5A and Table 4) and were encoded to the Cytoscape STRING database to remove singletons and the further ranking of OP-related hub genes (Fig. 5B), node degree calculation (Fig. 5C) and STRING pathway enrichment analysis (Fig. 5D) were performed.

**Table 4:** Information on overlapping target proteins of GCs and OP.

Number	HGNC Gene ID	Gene Symbol	Protein Name
1	1119	*BST2*	bone marrow stromal cell antigen 2 (*BST2*)
1	1932	*CHI3L1*	chitinase 3 like 1 (*CHI3L1)*
1	21184	*FNDC1*	fibronectin type III domain containing 1 (*FNDC1*)
1	5397	*IFI27*	interferon alpha inducible protein 27 (*IFI27*)
1	4054	*IFI6*	interferon alpha inducible protein 6 (*IFI6*)
1	5966	*IL11*	interleukin 11 (*IL11*)
1	7155	*MMP1*	matrix metallopeptidase 1 (*MMP1*)
1	10514	*SAA2*	serum amyloid A2 (*SAA2*)
1	10777	*SFRP2*	secreted frizzled related protein 2 (*SFRP2*)
1	11898	*TNFAIP6*	TNF alpha induced protein 6 (*TNFAIP6*)

### 3.6 Molecular docking studies

**Fig. 6:**
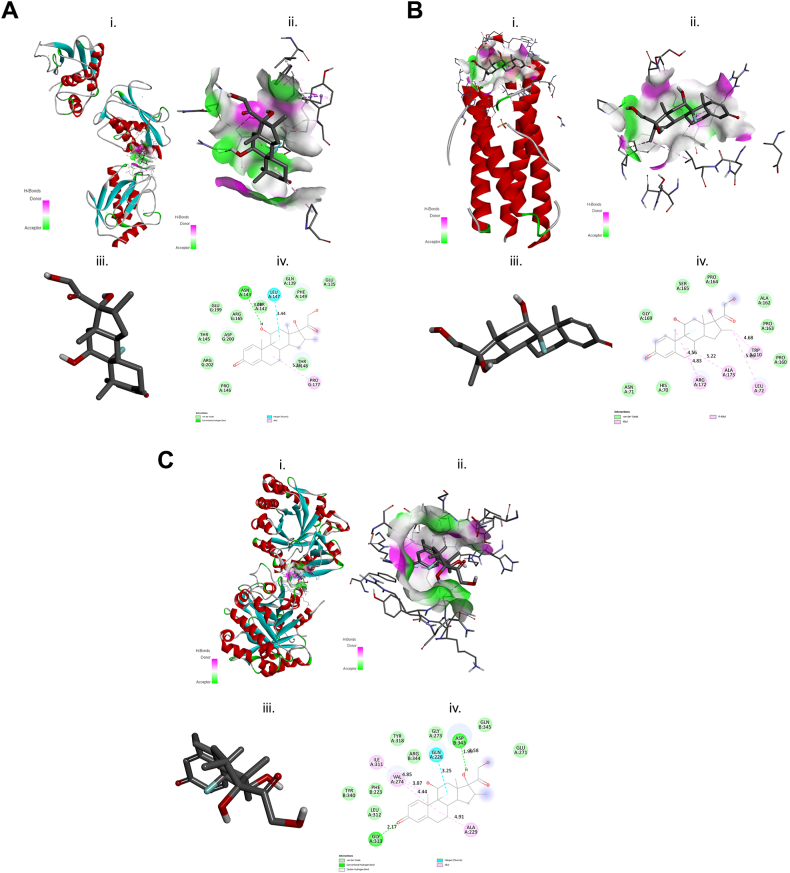
Schematic diagram of molecular docking of DEX and (A) matrix metallopeptidase 1 (MMP1), (B) interleukin 11 (IL11) and (C) chitinase 3 like 1 (CHI3L1); (i) ligand-receptor complex structure (ii) H-bond interacting atoms (iii) ligand (iv) ligand-receptor amino acid interactions.

**Fig. 7:**
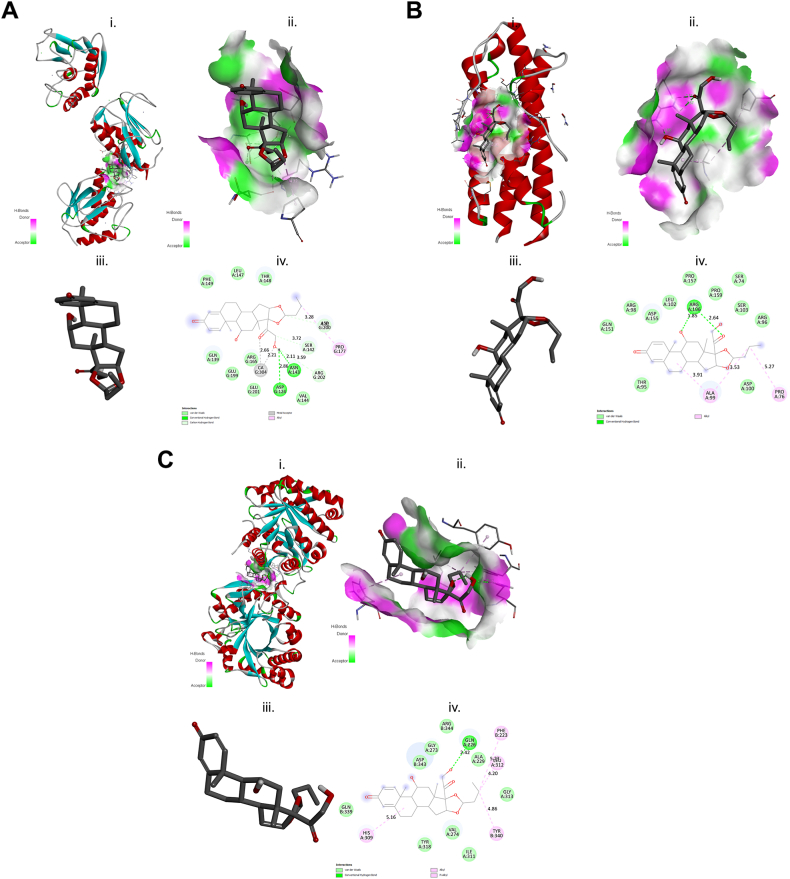
Schematic diagram of molecular docking of BDS and (A) matrix metallopeptidase 1 (MMP1), (B) interleukin 11 (IL11) and (C) chitinase 3 like 1 (CHI3L1); (i) ligand-receptor complex structure (ii) H-bond interacting atoms (iii) ligand (iv) ligand-receptor amino acid interactions.

**Fig. 8:**
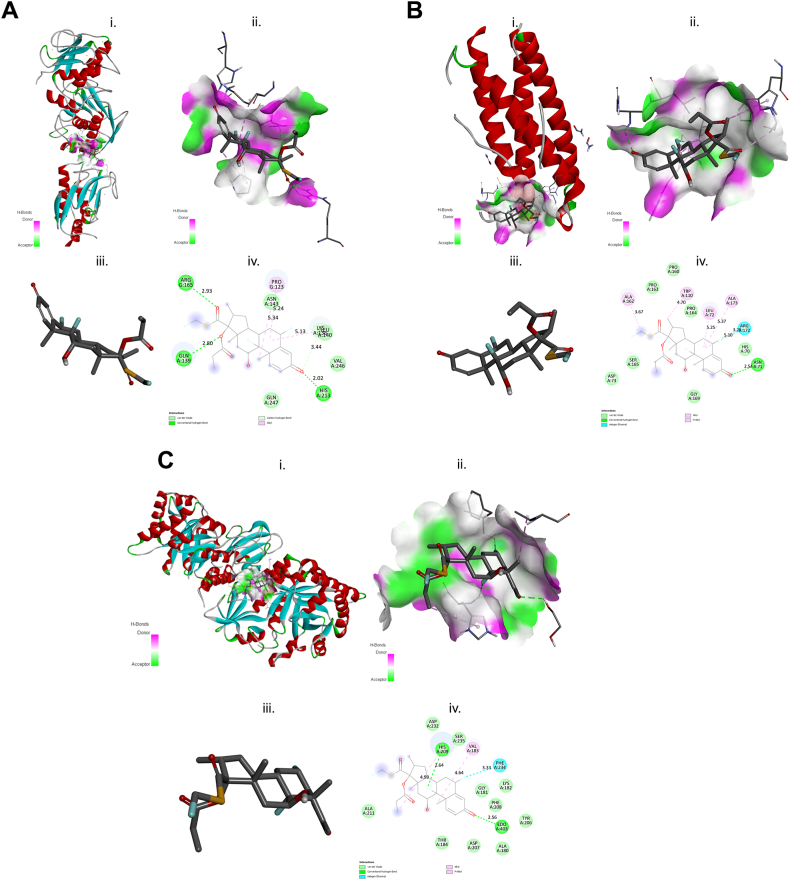
Schematic diagram of molecular docking of FLT and (A) matrix metallopeptidase 1 (MMP1), (B) interleukin 11 (IL11) and (C) chitinase 3 like 1 (CHI3L1); (i) ligand-receptor complex structure (ii) H-bond interacting atoms (iii) ligand (iv) ligand-receptor amino acid interactions.

Molecular docking studies were performed among the different structures of MMP1, CHI3L1, and IL11 with the three test GC compounds: DEX, BDS, and FLT (Fig. 4, Fig. 5, Fig. 6, iii.). This analysis is important to identify potential OP-inhibition and possible mechanism by interfering the OP-related transcriptional regulators. The summary of the highest binding affinities for each GC is shown in Table 5 and the detailed results are shown in Supplementary Table 3. The results were complex structure conformational changes and differences of MMP1, CHI3L1, and IL11 proteins on each GC compound (Fig. 6, Fig. 7, Fig. 8, i.); active binding pockets (Fig. 6, Fig. 7, Fig. 8; subsection ii); and different amino acid interaction (Fig. 6, Fig. 7, Fig. 8; subsection iv). The docking simulation for predicted OP-related genes on GC compound showed a highest binding affinity to CHI3L1, as it shows well fitted in the active binding site pocket with docking score of −9.4, −8.0, and −8.9 for DEX, BDS, and FLT, respectively (Fig. 6, Fig. 7, Fig. 8B, ii.). On the other hand, IL11 showed low docking score of −7.8 and −6.8 for DEX and BDS respectively.

**Table 5:** Summary of the binding energy (kcal mol^−1^) values of OP-related proteins with GCs compound.

Ligand-receptor complex	Energy (kcal/mol)
DEX-MMP1	−9.2
DEX-CHI3L1	−9.4
DEX-IL11	−7.8
BDS-MMP1	−7.3
BDS- CHI3L1	−8.0
BDS-IL11	−6.8
FLT-MMP1	−8.2
FLT- CHI3L1	−8.9
FLT-IL11	−8.8

## 4 Discussion

Many pathways play a role in the process of regulating osteogenesis and osteoporosis [39]. In order to fully understand the processes by which the whole system interacts with each other, whole genome expression studies have been done to better understand the underlying mechanisms. The osteogenic effect of DEX has been studied extensively due to its importance in causing secondary osteoporosis. Many papers have reported whole transcriptome response as microarray results for DEX [25,26,40]. Although FLT and BDS are used commonly, there are no established reports of osteoporotic effects related to their use. The study carried out the approach of conducting a microarray study on DEX on human mesenchymal stem cells while comparing it to FLT and BDS. With most reports done on upregulated genes and pathways in DEX treatment to promote osteogenic differentiation, this study focused on the downregulated genes and pathways involved for the three common glucocorticoids, respectively.

The reduction of this important biological process to osteogenesis may be a much bigger factor in the causation of osteoporosis. As shown in Table 2, *ADRA2A* receptor genes involved in leptin and other neurotransmitters are downregulated in all 3 compounds. It has been determined that animals lacking in leptin show significantly reduced bone density; while animals lacking in the leptin receptor show identical phenotypes [41]. The reduced expression of the *ADRA2A* receptor gene may help explain some of the osteoporotic properties of DEX.

Another interesting finding was the effect of DEX on multiple pathways involved in immune response (autoimmune thyroid disease, response to interferon-gamma, cellular response to interferon-gamma, regulation of expression of SLITs and ROBOs, immune response-activating cell surface receptor signaling pathway) and 2 pathways involved in the phagosome are downregulated as well (phagosome, ER-Phagosome pathway) (Table 3). The suppression of the immune system by DEX may play a role in inhibiting the osteogenic process and leading to secondary osteoporosis. With osteoclasts considered to be bone-immune cells [42], the effects of such pathways on the activity of osteoclasts will lead to an imbalance in the bone remodeling process which could promote osteoporosis. It was also found that GO:0001649 (Supplementary File 4) which is involved in osteoblast differentiation was also downregulated. Together with the negative effects on phagosome pathways and osteoblast differentiation, there is a clear imbalance in bone remodeling that could contribute to the occurrence of secondary osteoporosis. This information demonstrates the pathways that are downregulated and may lead to the occurrence of secondary osteoporosis which adds information to previous reports on the occurrence of osteoporosis without details in terms of involved pathways [43].

Though no report of bone loss has been reported in FLT treatment [44], various studies on the effects of FLT and BDS did not show bone loss. It was also found that in all glucocorticoids, COL14A1 has been downregulated. Although the importance of collagen synthesis for bone health is well known, the exact role of COL14A1 has not been determined [45]. Balla et al. have shown that COL14A1 was not downregulated in patients with OP indicating that it may have no role in the occurrence of the disease [46].

Several hub genes have been identified which may play important roles in osteogenesis and osteoporosis. BST2 regulates BMP-2 pathway and therefore any negative effects will disrupt the osteogenic process and potentially cause osteoporosis [47,48]. It was also shown that BST2 had positive correlation with osteogenic markers further indicating its down regulation role on osteoporotic potential [49]. CHI3L1 has shown important role in osteoclast differentiation, however, with contradictive findings. Wang et al. have shown that upregulation of CHI3L1 stimulated osteoclast differentiation and therefore contributed to the occurrence of osteoporosis [50]. On the other hand, Park et al. have found that CHI3L1 reduced osteoclastogenesis and therefore prevented bone resorption [51]. The contradictory finding might indicate an involvement of the gene; however, its role might be not directly involved with the osteoclastogenesis and could have an indirect effect. FNDC1 expression has been found to be associated with postmenopausal osteoporosis [52]. It was found that down regulation of FNDC1 was associated with disruption of autophagy [53]. Disruption of autophagy may lead to disruption of bone formation and therefore could lead to osteoporosis [54]. IFI6 and IFI27 were among the identified hub-gene. Both genes code for alpha interferons which have been identified to be involved in osteoporotic activity, yet their exact roles have not been identified yet [[55], [56], [57]]. IL-11 is a gene involved in the wnt signaling. Increase in wnt signaling enhances bone formation and therefore reduces osteoporotic potential [58]. Glucocorticoids reduce the activity of IL-11 and therefore inhibit osteoblast differentiation. MMP-1 is involved in the breakdown of collagen and therefore it disrupts the osteogenic process. For that reason, it is expected to play an important role in the occurrence of osteoporosis [59]. SAA2 is an immunomodulator that is found in the serum at high elevation after injury or inflammation and has been found that its increase blocks PTH-stimulated bone formation [60,61]. The significance of SAA2 downregulation needs to be further studied due to lack of sufficient information regarding this gene and its relationship to osteoporosis. SFRP2 is involved in maintaining normal skeletal development via modulation of the wnt signalling pathway [62]. It was found that SFRP2 knock out mice had reduced trabecular bone and in vitro studies showed poor osteogenic potential [62]. This indicates the importance of SFRP2 in normal bone formation but more work needs to be done to fully understand its role. TNFAIP6 has been found to disrupt osteogenic homeostasis when downregulated [63]. It was also found that TNFAIP6 was involved with aging related osteoporosis as its expression changes as the person get older [64,65]. These indicate the direct involvement of these genes in the process of osteoporosis. Although the exact role of every single hub-gene in the occurrences of osteoporosis in not fully clear, their direct involvement in normal bone formation has been identified. These findings warrant more studies that could look deeper into the roles of these hub genes in the occurrences of osteoporosis and the roles that dexamethasone, budesonide and fluticasone play on bone formation and therefore gain more insight into proper management for patients on chronic glucocorticoid therapy.

## 5 Conclusion

Our study focused on the downregulated gene expression profile of glucocorticoid compounds during osteogenic differentiation to determine the anti-osteoporotic potential in telomerase immortalized human bone marrow-derived mesenchymal stem cells. The result presented several genes were involved in bone remodeling, axon guidance, neurogenesis, immune response, and other related pathways, which potentially influence to further suppress the osteogenic process leading to secondary osteoporosis. The results suggested that budesonide and fluticasone propionate as potential treatments for osteoporotic patients, although further *in vivo* and detailed mechanism of action studies are still needed.

## Funding

The authors would like to thank the College of Dentistry Research Center and the Deanship of Scientific Research at 10.13039/501100002383King Saud University, Riyadh, Saudi Arabia for the funding of this research.

## CRediT authorship contribution statement

**Terrence Suministrado Sumague:** Writing – review & editing, Writing – original draft, Visualization, Validation, Software, Methodology, Investigation, Formal analysis, Data curation. **Abdurahman A. Niazy:** Writing – review & editing, Writing – original draft, Validation, Supervision, Resources, Project administration, Conceptualization. **Rhodanne Nicole A. Lambarte:** Writing – review & editing, Writing – original draft, Validation, Project administration, Investigation, Data curation, Conceptualization. **Ibrahim A. Nafisah:** Writing – review & editing, Software, Methodology, Data curation, Conceptualization. **Arief Gusnanto:** Writing – review & editing, Validation, Software, Methodology, Conceptualization.

## Declaration of competing interest

The authors declare that they have no known competing financial interests or personal relationships that could have appeared to influence the work reported in this paper.
