# Supplementary material for: Influence of budesonide and fluticasone propionate in the anti-osteoporotic potential in human bone marrow-derived mesenchymal stem cells via stimulation of osteogenic differentiation
Source: Heliyon. 2024 Oct 18;10(20):e39475. doi: 10.1016/j.heliyon.2024.e39475 (PMC11532851; doi:10.1016/j.heliyon.2024.e39475)
Supplement: Multimedia component 2 [file mmc2.docx]

List of intersection of GCs down-regulated and OP-associated genes. A total of 265, 37 and 469 in DEX, BDS and FLT respectively.

| **DEX** |
| --- |
| AAMP, ACAD9, ACKR3, ACTR1A, ADAMTSL2, ADRA2A, AGRN, AHSA1, AKR1A1, AKR1B1, AMPD2, AMT, ANPEP, ANXA6, APEH, APMAP, ARG2, ARL4C, ASS1, BAALC, BAP1, BCL2L11, BDKRB1, BGN, BMF, BSCL2, BST2, C1QTNF1, C1S, CA12, CA9, CCL5, CCNL2, CD2BP2, CDIPT, CDK5RAP2, CDK5RAP3, CFB, CFI, CH25H, CHD4, CHI3L1, CHID1, CHPF2, CHST3, CIB1, CLN3, CLU, CNIH3, COASY, COG4, COL14A1, COL5A1, COL6A2, CPE, CTNNA1, CTSA, CUL7, CXXC1, CYB561A3, DAG1, DANCR, DAP, DBP, DCTD, DGCR6L, DKK3, DMAP1, DNASE2, DPCD, ECH1, EDARADD, EDNRA, EEF1A1, EEF2, EHD3, EHMT2, EI24, EIF2B4, ELMOD3, ELOF1, ENO1, ENO2, ENPP2, EPHA2, EPHB2, ERGIC3, FAM20A, FBLN1, FDPSP2, FGF7, FIS1, FNDC1, FOXQ1, FRMD5, FTL, G6PC3, GALNT11, GAPDH, GAS6, GBA2, GHRH, GPER1, GPR108, GPR68, GPX4, GRB2, GRINA, GSTK1, HADHA, HDLBP, HLA-A, HLA-B, HLA-DMA, HLA-DMB, HLA-DPA1, HLA-DPB1, HLA-DRB1, HLA-DRB5, HM13, HSD17B14, HSP90AB1, ID3, IDH3B, IDH3G, IER3, IFI27, IFI35, IFI6, IFIT1, IFIT3, IL10RB, IL11, IL1R1, ILF3, ISG15, KCNN4, LDHA, LGALS9C, LINC01503, LMAN2, LRP1, LY6E, LZTR1, MADD, MAGED2, MAN1B1, MAP1A, MAPKAPK2, MARCKSL1, MFAP2, MMP1, MMP14, MMP7, MOB2, MRFAP1, MRGPRF, MRPL28, MX1, NDUFS2, NFIX, NKD2, NME4, NTHL1, NTM, NTMT1, NXF1, OLFML1, OLFML3, OXA1L, P4HB, PAFAH1B3, PARD3, PCOLCE, PEBP1, PGAM1, PGD, PHB, PHGDH, PI4KA, PIK3CD, PKIG, PKM, PLAUR, PLOD1, PLTP, POLD2, POR, POSTN, PPIB, PQBP1, PRDX2, PRKACA, PRKAG1, PRMT1, PRMT5, PRPF31, PSAP, PSMB8, PSMB9, PSMC3, PSMF1, PTGDS, PTPRU, RARRES2, RIC8A, RPL13A, RPL18, RPS3, RXRB, SAA2, SALL2, SCG5, SCMH1, SDC4, SDF2, SDHA, SERINC2, SERPING1, SFRP1, SFRP2, SIRT2, SLC38A5, SLC4A2, SLC6A8, SLCO4A1, SMPD1, SNAP47, SNRPB, SNRPN, SPAG7, ST3GAL1, ST6GALNAC6, SULF2, SYT7, TAX1BP3, THOC5, TIMP3, TMED3, TMED9, TMEM115, TMEM119, TMEM132A, TMEM140, TMEM208, TNC, TNFAIP6, TRO, TRPC4AP, TUBA4A, TXN2, UBA1, UBA7, UBL7, VAMP5, VPS8, WASF2, WDR6, WDR73, WDR74, XPC, ZMAT5, ZNF358, ZNF385D, ZY |
|  |
| **BDS** |
| ACKR3, ADAMTSL2, ADRA2A, ARL4C, BAALC, BDKRB1, BST2, CH25H, CHI3L1, CNIH3, COL14A1, CXCL12, FNDC1, FOXQ1, GPER1, ID2, IFI27, IFI6, IFIT1, IL11, KCNQ2, LGALS9C, MMP1, MMP7, MX1, PTGDS, RARRES2, RASD1, SAA2, SCG5, SEL1L3, SFRP2, SLC37A2, SLCO4A1, SULF2, TNFAIP6, TNFSF10 |
|  |
| **FLT** |
| AAMP, ABCA1, ABCC10, ABTB1, ACKR3, ACP2, ACP5, ACSF3, ADAM15, ADAMTSL2, ADD1, ADRA2A, AGT, AHSA1, AIP, AKR1A1, AKR1C1, AMPD2, AMT, ANKZF1, ANPEP, AP1B1, AP4M1, AP5Z1, APBB1, APEH, APMAP, APOE, ARAF, ARF1, ARG2, ARL4C, ARL6IP4, ARMC9, ARPC1B, ASPSCR1, ASS1, ATAD3B, AZIN2, B3GNT9, BAP1, BCAP31, BCL2L11, BCR, BGN, BMF, BMP2, BMP4, BRD9, BRINP1, BSCL2, BST2, C1QTNF1, C1S, C4B, CA12, CA9, CANT1, CAPN1, CAPS, CC2D1A, CCL7, CCND1, CCNL2, CD2BP2, CD81, CDIPT, CDK5RAP3, CEMIP, CENPB, CFB, CH25H, CHD4, CHI3L1, CHID1, CHMP4B, CHPF2, CHST14, CHST3, CIB1, CIRBP, CLCN7, CLN3, CLPTM1, CLU, CNIH3, CNPY3, COG4, COL14A1, COL5A1, COL6A2, COMT, CPSF1, CPTP, CPZ, CRIP2, CSF1, CSF2, CSK, CSNK1G2, CTDP1, CTSA, CTSD, CUL7, CXXC1, CYB561A3, CYBA, CYP46A1, CYTH2, DAGLB, DAP, DBP, DCTD, DEAF1, DGCR6L, DHX38, DKK3, DMAP1, DMKN, DNAJC4, DNASE2, DNM2, DOHH, DPP4, DVL1, E2F4, ECE1, ECH1, EDARADD, EDNRA, EEF2, EHD3, EHMT2, EI24, EIF2B4, EIF4G1, ELOF1, ENG, ENO1, ENO2, ESRRA, FADS3, FAM20A, FAM20C, FBLN1, FBXW5, FIS1, FNDC1, FOLR2, FOXQ1, FRMD5, FTL, FUS, G6PC3, G6PD, GAK, GALE, GALK1, GALNS, GALNT11, GAS6, GDF5, GGA1, GMDS, GMPPA, GNB2, GNPDA1, GOLGA6L9, GPAA1, GPER1, GPR108, GPX3, GPX4, GRINA, GSDMD, GTF3C1, GUSBP11, GYPC, HAS1, HDAC11, HDLBP, HERC2, HLA-B, HLA-DMB, HLA-DPA1, HM13, HMCN1, HOXB2, HPCAL1, HPS1, HRAS, HSD17B14, HSPB1, ID3, IDH3B, IER3, IFI27, IFI35, IFI6, IFIT3, IKZF1, IL10RB, IL11, IL17RC, IL1R1, IL6, INF2, INO80B, INPPL1, IRAK1, JMJD7, KCND2, KIAA0319L, KIF7, KIFC3, KLHDC8B, KLHL22, KRT18P55, KYNU, LAMA4, LAMB2, LAPTM4B, LIMK2, LINC01000, LINC01535, LMAN2, LONP1, LRP1, LRP10, LRPAP1, LRSAM1, LTB4R, LTBP3, LTBR, LY6E, LZTR1, MADD, MAEA, MAF1, MAFB, MAGED2, MAN1B1, MAN2B1, MAN2B2, MAP1LC3A, MAPK12, MAPK3, MAPKAPK2, MATN2, MCOLN1, MDFIC, MED12, MEN1, MFAP2, MFGE8, MFSD10, MGAT1, MICAL1, MLLT1, MLST8, MMP1, MMP14, MMP3, MOB2, MPG, MPPE1, MRFAP1, MRGPRF, MROH1, MRPL23, MRPL28, MTMR9LP, MXD4, MXI1, MYLIP, NAMPT, NBEAL2, NDUFS2, NHS, NISCH, NME4, NR1H2, NTHL1, NTMT1, NTN1, NUP214, OBSL1, OLFML3, ORAI2, ORM2, P4HA2, P4HB, P4HTM, PARD3, PAX8-AS1, PCOLCE, PDXK, PEBP1, PEMT, PEX6, PGAM1, PGS1, PHB, PHGDH, PHLDA3, PI4KA, PIGQ, PITHD1, PITPNM1, PITX1, PKIG, PLAUR, PLD3, PLOD1, PLXDC1, PLXNB2, PMAIP1, PMPCA, PNPLA6, POLD2, POMGNT2, POR, PPIB, PPP1CA, PPP1R15A, PQBP1, PRDX2, PRPF31, PSAP, PSMB9, PSMC3, PSMD3, PSMF1, PTGDS, PTPRU, PYCR1, RAB29, RAB40C, RARRES2, RASD1, RASGRF1, RBCK1, RBM19, RFNG, RHOD, RIC8A, RIN3, RIPK3, RNASET2, RNH1, RPL13A, RPL28, RPS19BP1, RPS2, RPS2P32, RRBP1, RRP12, RXRB, S1PR1, SAA2, SALL2, SCRN2, SDF4, SDHA, SEL1L3, SELO, SERINC2, SERPING1, SESN2, SFRP1, SFRP2, SGSM2, SH2B1, SH3BP5, SIRT2, SIRT6, SLC1A5, SLC22A17, SLC25A1, SLC27A4, SLC37A2, SLC38A5, SLC39A8, SLC3A2, SLC44A2, SLC4A2, SLC5A3, SLC6A8, SLC7A8, SLCO4A1, SMARCB1, SMARCD2, SMCR6, SMPD1, SNAP47, SNRNP70, SNRPB, SNRPN, SNTB2, SOBP, SPAG7, SPEG, SPG7, SPNS1, SQSTM1, SRM, ST3GAL1, ST3GAL4, ST6GALNAC6, SULF2, SUN2, SYT7, TAX1BP3, TBC1D17, TCEA2, TCN2, TGFB1, THAP11, THOC5, TIMM44, TMED3, TMED9, TMEM115, TMEM132A, TMEM140, TMEM203, TMEM208, TMEM63A, TNFAIP6, TNIP2, TRAPPC6A, TRMT2A, TRPC4AP, TSC2, TSPAN17, TUBA4A, TUBGCP2, TUBGCP6, TXN2, TYK2, UBA7, UBL7, UBXN6, ULK3, UNC45A, UNC93B1, UROS, USP19, VAC14, VEGFA, VGLL4, VPS39, WASF2, WBP2, WDR46, WDR6, WDR73, WDR74, XPC, XPO6, YPEL3, ZMAT3, ZMAT5, ZNF282, ZNF358, ZNF385D, ZNF503, ZNF513, ZRSR2 |
