# Supplementary material for: Influence of budesonide and fluticasone propionate in the anti-osteoporotic potential in human bone marrow-derived mesenchymal stem cells via stimulation of osteogenic differentiation
Source: Heliyon. 2024 Oct 18;10(20):e39475. doi: 10.1016/j.heliyon.2024.e39475 (PMC11532851; doi:10.1016/j.heliyon.2024.e39475)
Supplement: Multimedia component 3 [file mmc3.docx]

Molecular interactions of DEX, BDS, and FLT with amino acids of crystal structure of matrix metallopeptidase 1 (PDB: 3SHI), chitinase 3 like 1 (PDB: 8R41) and interleukin 11 (PDB: 4MHL) of *homo sapiens* to difference GCs

| **Ligand-receptor complex** | **Affinity kcal mol^-1^** | **Total H-Bonds** | **Total Bonds** | **Residue – Length (Interaction)** |
| --- | --- | --- | --- | --- |
| **DEX-MMP1** | -9.2 | 1 | 3 | ASN143 – 3.09 (H-Bond)  PRO177 – 5.36 (Alkyl)  LEU147 – 3.44 (Halogen)  ARG165 – (vdW)  ARG202 – (vdW)  ASP200 – (vdW)  GLU199 – (vdW)  THR145 – (vdW)  PRO146 – (vdW)  GLN139 – (vdW)  GLU135 – (vdW)  PHE149 – (vdW)  THR148 – (vdW) |
| **DEX-CHI3L1** | -9.4 | 2 | 7 | GLY313 – 3.09 (H-Bond)  ASP343 – 1.99 (H-Bond)  ASP343 – 3.58 (Carbon H-Bond)  ILE311 – 4.85 (Alkyl)  VAL274 – 4.44 (Alkyl)  ALA299 – 4.91 (Alkyl)  GLN226 – 3.25 (Halogen)  ARG344 – (vdW)  GLY273 – (vdW)  GLU199 – (vdW)  ASP200 – (vdW)  THR145 – (vdW)  ARG202 – (vdW)  PRO146 – (vdW)  THR148 – (vdW)  GLU135 – (vdW)  PHE149 – (vdW) |
| **DEX-IL11** | -7.8 | 0 | 5 | ARG172 – 4.83 (Alkyl)  ARG173 – 4.56 (Alkyl)  ALA173 – 5.22 (Alkyl)  LEU72 – 5.26 (Alkyl)  TRP110 – 4.68 (Alkyl)  ASN71 – (vdW)  HIS70 – (vdW)  PRO160 – (vdW)  PRO163 – (vdW)  ALA162 – (vdW)  PRO164 – (vdW)  SER165 – (vdW)  GLY169 – (vdW) |
|  |  |  |  |  |
| **BDS-MMP1** | -7.3 | 2 | 8 | ASN143 – 2.11 (H-Bond)  ASP124 – 2.86 (H-Bond)  ARG202 – 3.59 (Carbon H-Bond)  SER142 – 3.72 (Carbon H-Bond)  ASP200 – 3.28 (Carbon H-Bond)  CA304 – 2.66 (Metal-Acceptor)  CA304 – 2.21 (Metal-Acceptor)  PRO177 – 4.31 (Alkyl)  PH149 – (vdW)  THR148 – (vdW)  GLN139 – (vdW)  ARG165 – (vdW) |
| **BDS- CHI3L1** | -8.0 | 1 | 5 | GLN226 – 2.42 (H-Bond)  LEU312 – 5.23 (Alkyl)  HIS309 – 5.16 (Pi-Alkyl)  PHE223 – 5.23 (Pi-Alkyl)  TYR340 – 4.86 (Pi-Alkyl)  ALA229 – (vdW)  ARG344 – (vdW)  GLY273 – (vdW)  ASP343 – (vdW)  GLN339 – (vdW)  TYR318 – (vdW)  VAL274 – (vdW)  ILE311 – (vdW)  GLY313 – (vdW) |
| **BDS-IL11** | -6.8 | 2 | 5 | ARG106 – 1.85 (H-Bond)  ARG106 – 2.64 (H-Bond)  ALA99 – 391 (Alkyl)  ALA99 – 3.53 (Alkyl)  PRO76 – 5.27 (Alkyl)  ASP100 – (vdW)  THR95 – (vdW)  GLN151 – (vdW)  ARG98 – (vdW)  ASP155 – (vdW)  LEU102 – (vdW)  PRO157 – (vdW)  PRO159 – (vdW)  SER74 – (vdW)  SER103 – (vdW)  ARG96 – (vdW) |
|  |  |  |  |  |
| **FLT-MMP1** | -8.2 | 3 | 7 | ARG165 – 2.93 (H-Bond)  GLN139 – 2.80 (H-Bond)  HIS213 – 2.02 (H-Bond)  ASN143 – 5.34 (Carbon H-Bond)  LYS136 – 5.13 (Carbon H-Bond)  LEU140 – 3.44 (Carbon H-Bond)  PRO123 – 5.34 (Alkyl)  GLN247 – (vdW)  VAL246 – (vdW) |
| **FLT- CHI3L1** | -8.9 | 2 | 5 | HIS209 – 2.64 (H-Bond)  EDO403 – 2.56 (H-Bond)  PHE234 – 3.33 (Halogen)  VAL186 – 4.64 (Alkyl)  HIS209 – 4.99 (Pi-Alkyl)  ALA211 – (vdW)  THR184 – (vdW)  ASP207 – (vdW)  ALA180 – (vdW)  TYR206 – (vdW)  PHE208 – (vdW)  GLY181 – (vdW)  LYS182 – (vdW)  SER235 – (vdW) |
| **FLT-IL11** | -8.8 | 1 | 7 | ASN71 – 2.54 (H-Bond)  ARG172 – 3.25 (Halogen)  ALA173 – 5.37 (Alkyl)  TRP110 – 4.70 (Pi-Alkyl)  ALA162 – 3.67 (Pi-Alkyl)  LEU72 – 5.25 (Pi-Alkyl)  ARG172 – 5.10 (Pi-Alkyl)  ASP73 – (vdW)  SER165 – (vdW)  GLY169 – (vdW)  HIS70 – (vdW)  PRO164 – (vdW)  PRO160 – (vdW)  PRO163 – (vdW) |
